# Acid‐sensing ion channel 3 blockade inhibits durovascular and nitric oxide‐mediated trigeminal pain

**DOI:** 10.1111/bph.14990

**Published:** 2020-03-02

**Authors:** Christopher M. Holton, Lauren C. Strother, Isaac Dripps, Amynah A. Pradhan, Peter J. Goadsby, Philip R. Holland

**Affiliations:** ^1^ Headache Group, Basic and Clinical Neuroscience, Institute of Psychiatry, Psychology and Neuroscience King's College London London UK; ^2^ Department of Psychiatry University of Illinois at Chicago Chicago Illinois

## Abstract

**Background and Purpose:**

There is a major unmet need to develop new therapies for migraine. We have previously demonstrated the therapeutic potential of the acid‐sensing ion channel (ASIC) blockade in migraine, via an ASIC1 mechanism. ASIC3 is expressed in the trigeminal ganglion and its response is potentiated by NO that can trigger migraine attacks in patients. Thus we sought to explore the potential therapeutic effect of ASIC3 blockade in migraine.

**Experimental Approach:**

To investigate this, we utilised validated electrophysiological and behavioural rodent preclinical models. In rats, ASIC3 blockade using APETx2 (50 or 100 μg·kg^−1^, i.v.) was measured by using durovascular and NO‐evoked trigeminal nociceptive responses along with cortical spreading depression models. In mice, we sought to determine if periorbital mechanical sensitivity, induced by acute nitroglycerin (10 mg·kg^−1^, i.p.), was attenuated by APETx2 (230 μg·kg^−1^, i.p.), as well as latent sensitisation induced by bright light stress in a chronic nitroglycerin model.

**Key Results:**

Here, we show that the ASIC3 blocker APETx2 inhibits durovascular‐evoked and NO‐induced sensitisation of trigeminal nociceptive responses in rats. In agreement, acute and chronic periorbital mechanosensitivity induced in mice by nitroglycerin and subsequent bright light stress‐evoked latent sensitivity as a model of chronic migraine are all reversed by APETx2.

**Conclusion and Implications:**

These results support the development of specific ASIC3 or combined ASIC1/3 blockers for migraine‐related pain and point to a potential role for ASIC‐dependent NO‐mediated attack triggering. This has key implications for migraine, given the major unmet need for novel therapeutic targets.

**Abbreviations**
ASIC: acid‐sensing ion channel
BLS: bright light stress
CSD: cortical spreading depression
MMA: the middle meningeal artery
NTG: nitroglycerin
SNP: sodium nitroprusside
TNC: trigeminal nucleus caudalis

**What is already known**

- Acid‐sensing ion channel (ASIC) is a potential therapeutic target for migraine, likely via ASIC1.

**What this study adds**

- ASIC3 blockade with APETx2 inhibits durovascular‐evoked trigeminal nociceptive processing.
- APETx2 inhibits trigeminal sensitisation induced by NO donors that are known to trigger migraine clinically.

**What is the clinical significance**

- ASIC3 or combined ASIC1/3 blockers represent potential therapies for migraine.
- NO‐mediated migraine triggering may be in part ASIC‐dependent.

## 1 INTRODUCTION

Migraine is a severe disabling brain disorder (Stovner et al., 2018) characterised by bouts of unilateral pain resulting from activation of trigeminal sensory neurons and sensitisation of nociceptive processing (Goadsby et al., 2017). Preclinically, https://www.guidetopharmacology.org/GRAC/LigandDisplayForward?ligandId=2509 donors induce a delayed cutaneous allodynia‐like phenotype in rodents (Bates et al., 2010) in conjunction with increased trigeminal neuronal activity and hypersensitivity to intracranial and extracranial sensory stimulation (Akerman et al., 2019). Clinically, exposure to https://www.guidetopharmacology.org/GRAC/LigandDisplayForward?ligandId=7053 (NTG) an “NO donor” produces a transient headache (Ashina, Hansen, á Dunga, & Olesen, 2017) and the occurrence of migraine premonitory symptoms in healthy volunteers (Afridi, Kaube, & Goadsby, 2004). In migraineurs, it produces delayed migraine‐like attacks (Ashina et al., 2017) and triptan‐responsive cranial allodynia (Akerman et al., 2019) . While vasodilation may contribute to the acute headache, alternate mechanisms are likely to be involved in the delayed migraine‐like attacks (Marone et al., 2018). However, the mechanisms that lead to delayed hyperalgesia remain to be fully characterised. A greater understanding of which is required to help elucidate how individual migraine attacks are initiated and this will aid the development of novel therapeutic targets.

Sensory neurons expressing https://www.guidetopharmacology.org/GRAC/FamilyDisplayForward?familyId=118 (ASICs) convey nociception during several pain states in response to decreased extracellular pH (Ugawa et al., 2002; Yan et al., 2011; Yan, Wei, Bischoff, Edelmayer, & Dussor, 2013). https://www.guidetopharmacology.org/GRAC/ObjectDisplayForward?objectId=686 is the most sensitive ASIC to physiologically decrease pH (Deval et al., 2008) and, as such, may play a critical role in the initial phases of trigeminal sensitisation. It is co‐expressed with https://www.guidetopharmacology.org/GRAC/LigandDisplayForward?ligandId=695 (CGRP) in the rat trigeminal ganglion (Ichikawa & Sugimoto, 2002), where decreased pH results in CGRP release (Durham & Masterson, 2013). Pharmacologically the anti‐migraine therapeutic agent, the 5‐HT_1B/1D_ agonist https://www.guidetopharmacology.org/GRAC/LigandDisplayForward?ligandId=54, also inhibits the activity of ASICs in the rat trigeminal ganglion (Guo et al., 2018), while an ASIC‐sensitive proton‐mediated mechanism for the release of CGRP has been demonstrated. Given the therapeutic utility of the triptans (Ong & De Felice, 2018) and targeted modulation of CGRP signalling (Goadsby et al., 2017), ASIC modulation may represent a novel target with important translational implications.

Importantly, ASIC3 is potentiated by NO donors and nitroglycerin (NTG) increases acid‐evoked pain in humans (Cadiou et al., 2007). In agreement with a role for ASICs in migraine, we and others have previously identified the anti‐migraine efficacy of targeting specific ASICs in several validated preclinical models (Holland et al., 2012; Verkest et al., 2018; Wang et al., 2018; Yan et al., 2011).

Given the emerging role for ASICs in migraine (Karsan, Gonzales, & Dussor, 2018), the expression of ASIC3 in the trigeminal ganglion (Ichikawa & Sugimoto, 2002) and the trigeminal nucleus caudalis (TNC; Wang et al., 2018) along with the enhancement of ASIC3 activity by NO donors (Cadiou et al., 2007), we sought to determine the role of ASIC3 in migraine and further determine if NO‐induced hyperalgesia may be in part ASIC3‐dependent. We report that durovascular‐evoked and NO‐induced sensitisation of trigeminal nociceptive responses in the trigeminal nucleus caudalis are inhibited by ASIC3 blockade. We further demonstrate that nitroglycerin‐evoked delayed cutaneous allodynia in mice is attenuated by ASIC3 blockade. Finally, ASIC3 blockade reverses the delayed cutaneous allodynia evoked by environmental bright light stress |(BLS) in an nitroglycerin‐mediated preclinical model of chronic migraine (Kopruszinski et al., 2017; Tipton, Tarash, McGuire, Charles, & Pradhan, 2016). As such, ASIC3 may play a key role in migraine pathophysiology and NO‐induced delayed migraine in patients, highlighting further the potential of targeted modulation of ASICs and the future development of mambalgins (peptides found in the venom of the black mamba) for migraine.

## 2 METHODS

All procedures were conducted according to the Animals (Scientific Procedures) Act (1986), ethically approved by local animal welfare and ethical review bodies. Animal studies are reported in compliance with the ARRIVE guidelines (Kilkenny, Browne, Cuthill, Emerson, & Altman, 2010) and with the recommendations made by the *British Journal of Pharmacology.* In total, 43 male adult Sprague Dawley rats (280–315 g) and 36 adult male C57Bl6/J mice (20–30 g; Charles River, UK) were included in the study. Animals were grouped and housed in standard cages in climate‐controlled rooms with a 12‐hr light/dark cycle (07:00–19:00) and food and water were provided ad libitum.

### 2.1 General surgical set‐up

On the day of the surgery rats (*n* = 43) were initially anaesthetised with isoflurane (IsoFlo, 5%, Abbott, UK (RRID:SCR_010477)) and maintained with 1.5–2%. Following cannulation of the left femoral artery and both femoral veins the animals were switched to a continuous intravenous propofol infusion (PropoFlo, 33–50 mg·kg^−1^·hr^−1^, Abbott, UK). The additionally cannulated vein and artery were then used for the administration of test substances and to continuously monitor BP, respectively. Anaesthetic depth was confirmed by the lack of a withdrawal response or gross fluctuations in BP to noxious pinching of the hind paw. Following cannulation, rats were tracheotomised and ventilated with oxygen‐enriched air, with end‐tidal CO_2_ continuously monitored and ventilation adjusted as required to maintain normal physiological parameters (3.5–4.5%). Rectal temperature was maintained at 36.5–37°C via a rectal probe connected to a heating pad.

Following this initial surgery, rats were placed in a stereotaxic frame and underwent one of the following surgical preparations and experimental procedure as detailed below.

### 2.2 Neuronal recording in the trigeminal nucleus caudalis

To assess trigeminal nociceptive responses, the parietal bone was thinned to access the dura mater overlying the middle meningeal artery (MMA) and the area was covered in mineral oil to prevent drying. To access the trigeminal nucleus caudalis (TNC), a partial laminectomy of the first cervical vertebra was performed and the dura mater was opened to expose the caudal medulla. After completion of the surgery, animals were left to stabilise for at least 30 min before recording.

Stimulation of perivascular afferents of the trigeminal nerve was performed by placing a bipolar stimulating electrode on the dura mater adjacent to the middle meningeal artery. Dural nociceptive neurons in the trigeminal nucleus caudalis were identified via electrical stimulation (8–15 V, 0.5 Hz, 0.3–0.5 ms, 20 square wave electrical pulses) of the dura mater. Tungsten microelectrodes (0.5–1 MΩ) were carefully lowered into the trigeminal nucleus caudalis and used to record extracellularly from neurons, activated by dural electrical stimulation and with cutaneous facial receptive fields in the ophthalmic dermatome. The signal was amplified, filtered and recorded as previously described (Vila‐Pueyo, Strother, Kefel, Goadsby, & Holland, 2019).

When a cluster of wide dynamic range neurons sensitive to stimulation of the ophthalmic dermatome of the trigeminal nerve was identified, it was tested for convergent input from the dura mater. Trains of 20 stimuli were delivered at 5‐min intervals to assess the baseline response to dural electrical stimulation. Responses were analysed using post‐stimulus histograms with a sweep length of 100 ms and a bin width of 1 ms. When stable baseline values of the stimulus‐evoked responses were achieved (average of three stimulation series), responses were tested for up to 60 min following physiological intervention. For studies involving https://www.guidetopharmacology.org/GRAC/LigandDisplayForward?ligandId=9533 (SNP) facial receptive field characterisation consisted of 10 brush strokes applied to the facial receptive field over 7–8 s for the innocuous response and a pinch with forceps for 5 s for the noxious response. The change in cell firing from baseline at 50 after initial intervention as stated. Spontaneous activity (spikes·s^−1^) was recorded throughout and measures for analysis consisted of 60‐s epochs at baseline and then every 10 min. Post and peri‐stimulus time histograms of neural activity were displayed and analysed using Spike2 v8.

Following the establishment of stable baseline neuronal responses, rats (18 neuronal clusters from *n* = 16 rats) were administered either vehicle (0.5 ml normal saline) or https://www.guidetopharmacology.org/GRAC/LigandDisplayForward?ligandId=4135 (Alomone Labs, Israel) at 50 or 100 μg·kg^−1^ (*n* = 6 neuronal clusters per group) and durovascular‐evoked neuronal responses and spontaneous trigeminal nucleus caudalis neuronal activity recorded for 60 min.

To explore the impact of APETx2 on NO donor‐induced trigeminal neuronal sensitisation, rats (28 neuronal clusters from *n* = 22 rats) were assessed. Following the establishment of stable baseline neuronal responses, rats received one of the four treatment protocols (*n* = 7 neuronal clusters per group): saline/saline; APETx2 100 μg·kg^−1^/saline; saline/sodium nitroprusside 60 μg·kg^−1^ or APETx2 100 μg·kg^−1^/sodium nitroprusside 60 μg·kg^−1^. The impact of APETx2 on noxious and non‐noxious stimulation of the periorbital receptive field was conducted on a subset of the above rats (*n* = 5 per group).

Saline and APETx2 were administered as a slow intravenous bolus and sodium nitroprusside as a slow intravenous infusion (4 μg·kg^−1^·min^−1^) over 15 min that resulted in a transient sensitisation over the infusion period that returned to baseline within 10 min of cessation. Sodium nitroprusside‐induced neuronal sensitisation was recorded immediately following the cessation of acute sodium nitroprusside infusion, 50 min post APETx2 or vehicle control.

### 2.3 Cortical spreading depression recording

In a separate cohort of rats to assess cortical spreading depression induction (*n* = 5), a cranial window of approximately 2 × 2 mm was drilled in the parietal bone using a saline‐cooled drill and the underlying dura mater was carefully removed. This area was used for the insertion of a single needle 500 μm into the cortex to induce a cortical spreading depression event. Posterior to bregma, a similar opening was drilled in the parietal bone. In this area, a glass pipette with a tip diameter of 10 μm filled with 3‐M NaCl was placed 500 μm below the cortical surface for cortical steady state potential recording (direct current [DC] shift). The pipette was connected to an Ag/AgCl pellet electrode and an Ag/AgCl reference electrode that was placed subcutaneously in the neck. The electrode was connected to a headstage and the signal was amplified, filtered and displayed in a personal computer as previously described (Holland et al., 2012). To ensure reliable cortical spreading depression induction, two control cortical spreading depressions were initiated, the second of which occurred 5 min following the administration of vehicle control. Following an appropriate refractory period, rats were then administered APETx2 at 100 μg·kg^−1^ and subsequent cortical spreading depression inductions were conducted at 30 and 70 min post APETx2.

### 2.4 NO induced periorbital mechanical hypersensitivity

To further assess the efficacy of ASIC3 blockade on NO donor‐induced trigeminal sensitisation, we utilised two different models of nitroglycerin (NTG)‐evoked periorbital mechanical hypersensitivity in mice. Facial mechanosensitivity was assessed via von Frey filament application to the periorbital region of the face. To do so, animals were acclimatised to the testing apparatus at least 1 day before and again 1 hr prior to testing. The testing apparatus consisted of individual ventilated acrylic enclosures (6 × 6 cm) placed on a self‐standing perforated metal platform (Ugo Basile). To facilitate facial sensory testing, mice were additionally habituated to a 4‐oz paper cup, placed inside the acrylic enclosure. (Tipton et al., 2016). Facial mechanical sensitivity was then tested with the mouse freely moving within the up‐right paper cup that permitted unhindered access to the periorbital region, caudal to the eyes and near the midline. Graduated von Frey filaments were then applied to the periorbital region starting with the 0.4‐g filament using the up–down method to calculate mechanical withdrawal thresholds (Chaplan, Bach, Pogrel, Chung, & Yaksh, 1994).

In the acute study, mice (*n* = 12) were tested at baseline and following 2 hr post nitroglycerin treatment (10 mg·kg^−1^, i.p.). To minimise the number of mice and in keeping with the ARRIVE guidelines, mice were retested following 1 week. Mice received two doses of APETx2 (0.23 mg·kg^−1^, i.p.) and a single dose of nitroglycerin (10 mg·kg^−1^, i.p.), and periorbital withdrawal thresholds were assessed 2 hr post nitroglycerin. Due to its short half‐life, APETx2 was administered 30 min before nitroglycerin treatment and again 30 min before sensory threshold testing.

To assess the efficacy of APETx2 to block NO donor‐induced trigeminal sensitisation in a more chronic paradigm, we adapted a rodent model of triptan‐induced latent sensitisation (Kopruszinski et al., 2017). In this model, rodents exposed to persistent sumatriptan exposure that is subsequently withdrawn develop a basal periorbital mechanical hypersensitivity that normalises following triptan withdrawal. However, rodents previously exposed to sumatriptan but not vehicle control demonstrate a “latent sensitisation” whereby exposure to a presumed migraine trigger (bright light stress) reinstates the periorbital mechanical hypersensitivity. Persistent exposure to nitroglycerin, like sumatriptan, is also known to induce a basal periorbital mechanical hypersensitivity (Tipton et al., 2016) and as such, we hypothesised that bright light stress‐induced latent sensitisation was also possible following chronic nitroglycerin administration and subsequent recovery and, furthermore, that ASIC3 blockade could modulate this. To test this hypothesis, baseline periorbital mechanosensitivity was assessed in a separate cohort of mice (*n* = 24), which were then divided into three groups that were randomly assigned to receive chronic vehicle control followed by APETx2 prior to bright light stress, chronic nitroglycerin followed by acute administration of vehicle control prior to bright light stress or chronic nitroglycerin followed by acute administration of APETx2 prior to bright light stress. After baseline assessment, one animal was excluded due to a consistently low mechanical sensitivity threshold and as such, the final analysis was conducted on *n* = 7, 8 and 8 mice, respectively. Vehicle (0.9% saline, i.p.) and nitroglycerin (10 mg·kg^−1^ NTG, i.p.) were administered on Days 1, 3, 5, 7 and 9, with mechanosensitivity assessed on Days 1, 5 and 9 both before (basal) and 2‐hr post administration (Tipton et al., 2016). After the establishment of a basal hyperalgesia (Day 9), mice were allowed to recover for at least 1 week until basal mechanosensitivity returned to baseline levels. All mice were then exposed to 1 hr of bright light stress (3,000–3,500 lux) on Days 17 and 18 as previously reported (Kopruszinski et al., 2017). On Day 18, mice were treated with APETx2 (0.23 mg·kg^−1^) or vehicle (0.9% saline, i.p.) 30 min before and again 30 min post bright light stress, and periorbital mechanosensitivity was assessed 2‐hr post bright light stress as detailed in Figure 3d.

### 2.5 Data and statistical analysis

**Figure 1:**
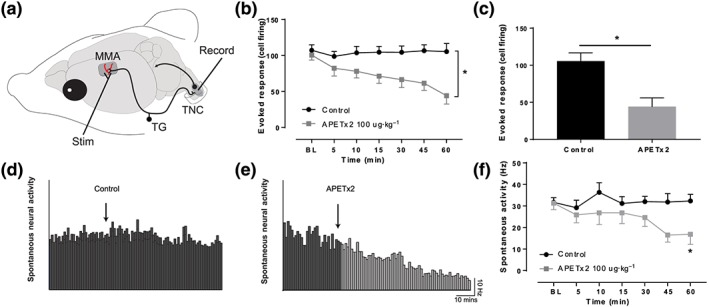
APETx2 reduces durovascular nociceptive‐evoked and spontaneous neuronal activity in the trigeminal nucleus caudalis (TNC). Experimental set‐up in the rat (a). Durovascular nociceptive afferents arising in the trigeminal ganglion (TG) are activated via stimulation of the dura mater surrounding the middle meningeal artery (MMA). Durovascular‐evoked responses are then recorded in the TNC. TNC durovascular nociceptive‐evoked neuronal responses are significantly reduced following APETx2 (b), starting from 45 min post infusion and remain significantly reduced at 1 hr (c). Examples of TNC spontaneous neuronal activity following control (d) and APETx2 infusion (e) that was significantly decreased over the 1‐hr recording window in APETx2 (f), but not vehicle control treated rats compared to baseline. *P < .05, *n* = 6 neuronal clusters per group from *n* = 5 rats per group [Colour figure can be viewed at http://wileyonlinelibrary.com]

**Figure 2:**
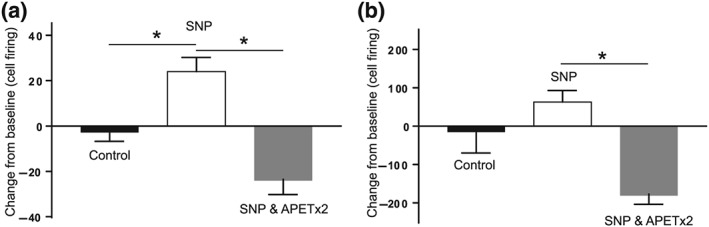
APETx2 blocks NO‐induced sensitisation to noxious stimuli. There was a significant overall difference in durovascular‐evoked trigeminal nucleus caudalis neuronal activation across all groups. Sodium nitroprusside (SNP) induced increased durovascular‐evoked responses (a) when compared to vehicle control treated rats. Pretreatment with APETx2 significantly reduced the SNP‐induced increase in durovascular‐evoked responses (a). There was a significant overall difference in noxious pinch‐evoked trigeminal nucleus caudalis neuronal activation (b) across all groups. Sodium nitroprusside induced a non‐significant modest increase in noxious pinch‐evoked responses (b) when compared to vehicle control treated rats. Pretreatment with APETx2 significantly reduced the noxious pinch‐evoked responses when compared to SNP treated rats (b). *P < .05; *n* = 7/5 neuronal clusters per group from 22 and 20 rats for (a) and (b), respectively

**Figure 3:**
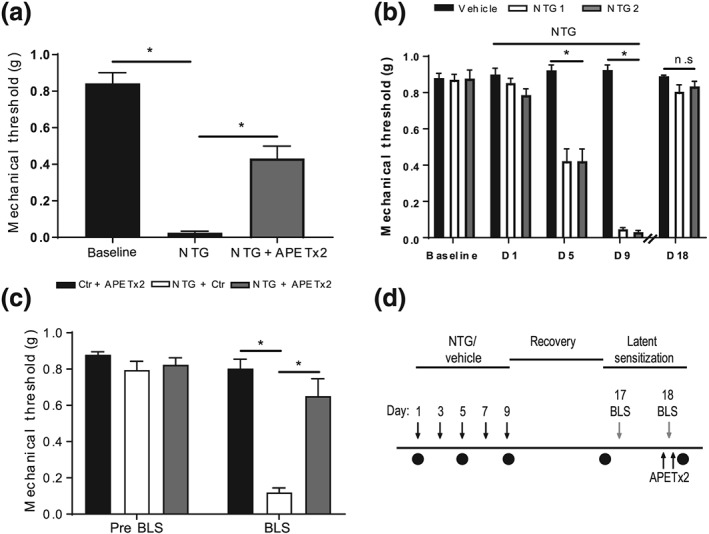
APETx2 inhibits nitroglycerin‐evoked periorbital mechanosensitivity in mice. There was a significant overall difference in periorbital mechanosensitivity across all groups (a). Acute nitroglycerin (NTG) induced a decreased periorbital mechanosensitivity in mice that was partially reversed by pretreatment with APETx2. Chronic NTG administration in mice (NTG‐primed) produced a basal periorbital mechanosensitivity (b) that reached significance from Day 5 when compared to vehicle control mice. Following withdrawal of NTG periorbital mechanosensitivity returned to that of the non‐sensitised vehicle control group. Subsequent exposure to bright light stress (BLS) resulted in a significant overall difference in periorbital mechanosensitivity (c). NTG‐primed mice demonstrated an increased periorbital mechanosensitivity when compared to vehicle control and APETx2 treated mice. The periorbital mechanosensitivity evoked in response to BLS was blocked by pretreatment with APETx2, returning to that of vehicle control and APETx2 treated mice. Timeline of latent sensitisation to BLS protocol (d). Animals were sensitised with chronic administration of NTG (10 mg·kg^−1^, i.p.) every second day for 9 days. Periorbital mechanical sensitivity was assessed with the von Frey assay (black circles) before and 2‐hr post NTG treatment on Days 1, 5, and 9. Animals were recovered for 1 week and then on Day 17 mechanical sensitivity was reassessed prior to 1 hr of BLS. On Day 18, animals were treated with either APETx2 (0.23 mg·kg^−1^) or vehicle control 30 min prior to and again 30 min post BLS. Mechanical sensitivity was then assessed 2‐hr post BLS. *P < .05; *n* = 12 mice for (a) and *n* = 7/8/8 mice per group for (b) and (c). For (b), NTG 1 and NTG 2 represent NTG‐primed mice that subsequently received vehicle control or APETx2, respectively in (c). One mouse was excluded from the control group due to ill health

The data and statistical analysis comply with the recommendations of the *British Journal of Pharmacology* on experimental design and analysis in pharmacology (Curtis, Ashton, Moon, & Ahluwalia, 2018) and were performed using GraphPad (RRID:SCR_002798) or SPSS (RRID:SCR_002865), in agreement with *BJP* guidelines. All studies were designed to generate groups of equal size, and experimental animals or groups (behavioural analysis) were randomly assigned to treatment groups before being analysed blind to the experimental group. For behavioural experiments, this consisted of initially dividing the mice into four equal groups to minimise baseline variances, and the four groups were then randomly assigned to control or experimental compounds. One mouse was subsequently excluded from the behavioural analysis as it showed signs of uncontrollable distress and was immediately and humanely culled. Therefore, in Figure 3b,c, group sizes are *n* = 7, 8 and 8 for control, nitroglycerin and nitroglycerin plus APETx2, respectively. All statistical analysis was conducted on group sizes of at least *n* = 5. For behavioural analysis, the group size reported is the number of independent values (mice), and statistical analysis was conducted on these independent values. For in vivo electrophysiological studies to minimise animal use and only where stable recordings existed following testing of a vehicle control, a small proportion of rats were then randomly assigned to receive one of the experimental compounds/doses. To ensure that these were not technical replicates, an entirely new neuronal cluster was identified, and as such, the results contain the following biological replicates. In Figure 1a,b the results reported are from *n* = 12 neuronal clusters in *n* = 10 rats, while in Figure 2a the results reported are from *n* = 28 neuronal clusters in 22 rats. Sample sizes are based on previous studies (Holland et al., 2012; Tipton et al., 2016) and sample size calculations with an estimated effect size = 20–40%, probability = 0.05 and power = 0.8–0.9 calculated using G.power software.

For graphical purposes, data are presented as mean ± standard error and all statistical analysis was conducted on raw data tested for homogeneity of sample variance (where appropriate) using the Levene's test. A level of probability of *P* ≤ .05 was defined as the threshold for statistical significance and where appropriate, post hoc tests were only conducted following a significant ANOVA to protect against Type 1 errors. The impact of APETx2 on evoked‐trigeminal‐neuronal responses was assessed via a mixed model two‐way ANOVA with Sidak's multiple comparisons, compared to the vehicle control treated rats. The impact of APETx2 on spontaneous trigeminal‐neuronal firing was assessed via a repeated measures (RM)‐ANOVA, compared to baseline firing rates. The impact of APETx2 on sodium nitroprusside‐evoked trigeminal sensitisation was assessed via one‐way ANOVA with Sidak's multiple comparison test, comparing the change in cell firing from baseline at the 50‐min time point. For behavioural analysis, the impact of APETx2 on acute nitroglycerin ‐evoked mechanical sensitivity was assessed via RM‐ANOVA with Sidak's multiple comparison test. Changes in basal mechanical sensitivity in response to chronic nitroglycerin were assessed via mixed model two‐way ANOVA with Sidak's multiple comparison test. Subsequent analysis of the effect of bright light stress and APETx2 on nitroglycerin‐primed mice was assessed via one‐way ANOVA with Sidak's multiple comparison test as detailed in Table S1. Where appropriate, if Mauchly's test of sphericity was violated, appropriate corrections to degrees of freedom according to Greenhouse–Geisser were made.

### 2.6 Materials

APETx2 (Alomone Labs, Israel) was dissolved in water to 500 μg·ml^−1^ and diluted in 0.9% saline. For rat studies, APETx2 was administered intravenously at 50 or 100 μg·kg^−1^, while for mice APETx2 was administered intraperitoneally at 0.23 mg·kg^−1^. Sodium nitroprusside (SNP; Sigma, UK) was dissolved in 0.9% saline immediately prior to use, with 60 μg·kg^−1^ infused intravenously over 15 min at 0.5 ml·hr^−1^. Nitroglycerin (NTG; Hospira, UK) was dissolved in 0.9% saline immediately prior to intraperitoneal injection at a final dose of 10 mg·kg^−1^.

### 2.7 Nomenclature of targets and ligands

Key protein targets and ligands in this article are hyperlinked to corresponding entries in http://www.guidetopharmacology.org, the common portal for data from the IUPHAR/BPS Guide to PHARMACOLOGY (Harding et al., 2018), and are permanently archived in the Concise Guide to PHARMACOLOGY 2019/20 (Alexander et al., 2019).

## 3 RESULTS

### 3.1 APETx2 reduces durovascular‐evoked nociceptive responses in the trigeminal nucleus caudalis

Durovascular nociceptive‐evoked trigeminal nucleus caudalis neuronal responses (Figure 1a) were significantly reduced following ASIC3 blockade with APETx2 100 μg·kg^−1^ (Figure 1b,c, *n* = 6 neuronal clusters per group in 10 rats) in the absence of any BP effects when compared to vehicle control treated rats. APETx2 also significantly reduced spontaneous trigeminal nucleus caudalis neuronal activity (Figure 1d–f, *n* = 6 neuronal clusters per group in 10 rats). APETx2 at 50 μg·kg^−1^ had no effect on any parameter tested (data not shown, *n* = 6 rats).

### 3.2 APETx2 reduces NO‐evoked nociceptive responses in the trigeminal nucleus caudalis

Intravenous infusion of the NO donor sodium nitroprusside resulted in a transient increase in evoked trigeminal nucleus caudalis neural activity (Figure 2a, *n* = 7 neuronal clusters per group in 22 rats). Pretreatment with APETx2 50‐min before sodium nitroprusside‐induced sensitisation inhibited sodium nitroprusside‐induced durovascular‐evoked firing (Figure 2a). Further, sodium nitroprusside resulted in a modest non‐significant increase in noxious pinch‐evoked responses from the periorbital region (Figure 2b, *n* = 5 neuronal clusters per group in 20 rats). Pretreatment with APETx2 significantly reduced the noxious pinch‐evoked responses when compared to sodium nitroprusside treated rats (Figure 2b). There was no impact of sodium nitroprusside on non‐noxious stimulation of the periorbital region and APETx2 alone had no significant impact on durovascular, noxious or non‐noxious evoked responses in the trigeminal nucleus caudalis (data not shown).

### 3.3 APETx2 does not inhibit mechanically‐induced cortical spreading depression

We have previously identified a potential ASIC1‐dependent inhibition of cortical spreading depression (Holland et al., 2012), however APETx2 has limited blood–brain barrier penetration, suggesting a potential lack of CNS actions. In agreement, with a likely peripheral action of APETx2 on durovascular‐evoked nociceptive responses, we did not observe any inhibition of mechanically induced cortical spreading depressions in all animals (data not shown, *n* = 5).

### 3.4 APETx2 reduces nitroglycerin‐evoked periorbital mechanosensitivity in mice

Acute nitroglycerin administration resulted in a significantly increased periorbital mechanosensitivity in mice. Pretreatment with APETx2 resulted in a significant decrease in this acute nitroglycerin‐induced periorbital mechanosensitivity that did not fully recover to baseline (Figure 3a, *n* = 12 mice).

In addition, we adapted a model of medication overuse “latent sensitisation” in mice (Kopruszinski et al., 2017), whereby rats systemically exposed to triptans demonstrate cutaneous allodynia that normalises following withdrawal (Kopruszinski et al., 2017). Despite normalised facial mechanosensitivity, these mice maintain a heightened response to presume migraine triggers such as exposure to bright light stress. Herein, we induced this latent sensitisation via chronic nitroglycerin exposure and following 1 week of withdrawal exposed mice to bright light stress. In agreement, exposure of mice to nitroglycerin for 9 days generated a periorbital mechanosensitivity when compared to vehicle control treated mice that normalised after 1 week withdrawal (Figure 3b, *n* = 23 mice). Subsequent exposure to bright light stress resulted in an increased periorbital mechanosensitivity in nitroglycerin‐primed mice but not nitroglycerin‐naïve mice (Figure 3c, *n* = 8 and 7 mice, respectively, as one mouse was excluded from the control group due to ill health). While nitroglycerin‐primed mice pretreated with APETx2 showed no periorbital mechanosensitivity (Figure 3c, *n* = 8 mice per group) when compared to nitroglycerin‐primed mice exposed to vehicle control.

## 4 DISCUSSION

Here, we demonstrate a clear translational potential for ASIC3 blockade in migraine. The ASIC3 blocker APETx2 inhibits both durovascular‐ and NO‐evoked trigeminal nociception in rats. Further, APETx2 inhibits nitroglycerin and bright light stress‐evoked cutaneous allodynia in nitroglycerin‐naïve and nitroglycerin ‐primed mice, respectively. Given the ability of NO donors such as nitroglycerin to trigger migraine attacks reliably in patients (Ashina et al., 2017) and cutaneous allodynia/trigeminal sensitisation in rodents (Akerman et al., 2019; Bates et al., 2010), our data suggest that this NO‐mediated effect may be at least in part be ASIC3‐dependent. ASIC3 is expressed on trigeminal ganglion neurons (Ichikawa & Sugimoto, 2002) and dural afferents (Yan et al., 2011), and its activation by decreased pH leads to elevated CGRP release (Durham & Masterson, 2013). Given that targeted CGRP therapies likely have a significant peripheral effect at the level of the trigeminal ganglion and represent the current state of the art therapeutics for migraine (Ong, Wei, & Goadsby, 2018), we propose herein that ASIC3 blockade may represent a potential adjunct target for reducing CGRP release. Importantly, we have previously demonstrated that amiloride, via an ASIC1 mechanism, showed similar beneficial effects on durovascular‐evoked trigeminal nociception (Holland et al., 2012). Specific ASIC1 blockade further inhibited cortical spreading depression propagation that we did not observe in the current study, likely due limited blood–brain barrier penetrability of APETx2, suggesting that the ASIC3‐mediated effects are likely peripheral at the level of the trigeminal afferents (Yan et al., 2013). Although specific central effects cannot be ruled out as ASIC3 is expressed centrally, including in the hypothalamus (Meng, Wang, Chen, Xu, & Zhou, 2009) and trigeminal nucleus caudalis, where its expression is up‐regulated in a dural‐inflammatory mediated preclinical model of migraine (Wang et al., 2018). In agreement with the potential efficacy of targeting ASIC signalling for migraine, it has recently been demonstrated that specific blockade of the ASIC1 subunit can inhibit NO‐evoked cutaneous allodynia in rats (Verkest et al., 2018), which supports our data and suggests that NO‐mediated attack triggering may have important central and peripheral actions on ASICs.

The preclinical models utilised herein have demonstrated clear translational validity (Holland et al., 2012; Kopruszinski et al., 2017; Tipton et al., 2016), including predicting clinical trial failure (Goadsby, Hoskin, & Knight, 1998). As such, we propose that an ASIC3 or combined ASIC1/3 blocker may prove beneficial for the treatment of migraine and that ASIC‐dependent mechanisms may in part underlie the increased susceptibility of migraineurs to NO donors.

## AUTHOR CONTRIBUTIONS

P.R.H, C.M.H, L.C.S and P.J.G initiated the project and designed all the experiments. C.M.H and P.R.H conducted the in‐vivo electrophysiology. L.C.S, I.D. and A.P conducted the behavioural study.

## CONFLICT OF INTEREST

The authors declare no conflicts of interest.

## DECLARATION OF TRANSPARENCY AND SCIENTIFIC RIGOUR

This Declaration acknowledges that this paper adheres to the principles for transparent reporting and scientific rigour of preclinical research as stated in the *BJP* guidelines for https://bpspubs.onlinelibrary.wiley.com/doi/full/10.1111/bph.14207, and https://bpspubs.onlinelibrary.wiley.com/doi/full/10.1111/bph.14206, and as recommended by funding agencies, publishers and other organisations engaged with supporting research.

## Supporting information

Table S1 Supporting Information
