# Supplementary material for: Acid‐sensing ion channel 3 blockade inhibits durovascular and nitric oxide‐mediated trigeminal pain
Source: Br J Pharmacol. 2020 Mar 2;177(11):2478–86. doi: 10.1111/bph.14990 (PMC7205795; doi:10.1111/bph.14990)
Supplement: Supplementary file 1 — Table S1 Supporting Information [file BPH-177-2478-s001.docx]

| **Figure** | **Analysis** | Time | Treatment | Interaction | Notes |
| --- | --- | --- | --- | --- | --- |
| 1B | Mixed model 2-way ANOVA | F_(6,60)_ = 5.25, *P*<0.05. | F_(1,10)_ = 8.35, *P*<0.05. | F_(6,60)_ = 5.62, *P*<0.05. | Control v’s APETx2 100 µgkg^-1^. |
| 1C | Sidaks post-hoc multiple comparisons |  | t_(9.65)_ = 3.46,  *P*< 0.05. |  | Control v’s APETx2 100 µgkg^-1^ at 45 mins. |
| 1C | Sidaks post-hoc multiple comparisons |  | t_(9.98)_ = 3.78,  *P*< 0.05. |  | Control v’s APETx2 100 µgkg^-1^ at 60 mins. |
| 1F | RM-ANOVA | F_(6, 30)_ = 6.55,  *P*< 0.05. |  |  | APETx2 100 µgkg^-1^. |
| 1F | RM-ANOVA | F_(6, 30)_ = 1.57,  *P*> 0.05. |  |  | Control. |
| 2A | One-way ANOVA |  | F_(3,24)_= 12.97, *P*< 0.05. |  | Overall effect. |
| 2A | Sidaks post-hoc multiple comparisons |  | **t_(24)_ = 3.28,**  ***P*< 0.05.** |  | Control v’s Sodium nitroprusside (SNP). |
| 2A | Sidaks post-hoc multiple comparisons |  | **t_(24)_ = 5.79,**  ***P*< 0.05.** |  | SNP v’s SNP and APETx2 100 µgkg^-1^. |
| 2B | One-way ANOVA |  | F_(3,16)_= 5.74,  *P*< 0.05. |  | Overall effect. |
| 2B | Sidaks post-hoc multiple comparisons |  | **t_(16)_ = 1.21,**  ***P*> 0.05.** |  | Control v’s Sodium nitroprusside (SNP). |
| 2B | Sidaks post-hoc multiple comparisons |  | **t_(16)_ = 3.93,**  ***P*< 0.05.** |  | SNP v’s SNP and APETx2 100 µgkg^-1^. |
| 3A | RM ANOVA |  | F_(1.6, 17.3)_ = 62.65, *P*< 0.05. |  | Overall effect. |
| 3A | Sidaks post-hoc multiple comparisons |  | **t_(11)_ = 14.72,**  ***P*< 0.05.** |  | Baseline v’s NTG 10 mgkg^-1^. |
| 3A | Sidaks post-hoc multiple comparisons |  | **t_(11)_ = 5.73,**  ***P*< 0.05.** |  | NTG 10 mgkg^-1^ v’s NTG10 mgkg^-1^ plus APETx2 100 µgkg^-1^.. |
| 3B | Mixed model two-way ANOVA | F_(2.8, 55.6)_ = 77.62,  *P*< 0.05. | F_(2, 20)_ = 65.06,  *P*< 0.05. | F_(8, 80)_ = 21.68,  *P*< 0.05 | Overall effect. |
| 3B | Sidaks post-hoc multiple comparisons |  | **t_(8.76)_ = 1.65,**  ***P*> 0.05.** |  | Control v’s NTG 10 mgkg^-1^ group 1. |
| 3B | Sidaks post-hoc multiple comparisons |  | **t_(9.59)_ = 1.31,**  ***P*> 0.05.** |  | Control v’s NTG 10 mgkg^-1^ group 2 . |
| 3C | One-way ANOVA |  | F_(2, 20)_ = 30.21,  *P*< 0.05. |  | Overall effect. |
| 3C | Sidaks post-hoc multiple comparisons |  | **t_(20)_ = 7.30,**  ***P*< 0.05.** |  | Control v’s NTG |
| 3C | Sidaks post-hoc multiple comparisons |  | **t_(20)_ = 5.87,**  ***P*< 0.05.** |  | NTG v’s NTG plus APETx2 |
| 3C | Sidaks post-hoc multiple comparisons |  | **t_(20)_ = 1.62,**  ***P*> 0.05.** |  | Control v’s NTG plus APETx2 |
